# Factors Affecting Psychological Resilience in First Responders and Rescue Team Members

**DOI:** 10.1192/j.eurpsy.2025.1824

**Published:** 2025-08-26

**Authors:** M. G. Goksu, N. S. Soral, Z. Sofuoglu

**Affiliations:** 1 Izmir Demokrasi Üniversitesi, Izmir, Türkiye; 2 Public Health, Izmir Demokrasi Üniversitesi, Izmir, Türkiye

## Abstract

**Introduction:**

Disasters, including natural catastrophes, accidents, and man-made events, impose significant challenges on communities and individuals. First responders, such as rescue teams, firefighters, and prehospital emergency medical services personnel, play a crucial role in mitigating these impacts. Beyond their physical capabilities, these individuals must possess high levels of psychological resilience to perform effectively under extreme and stressful conditions. Understanding the factors that influence this resilience is essential for improving their performance and well-being. This systematic review aims to identify and examine the factors that affect psychological resilience among first responders and rescue team members during disasters and emergencies. The goal is to provide a comprehensive understanding of how resilience can be supported and enhanced in these critical professionals.

**Objectives:**

To identify the key factors that influence psychological resilience in first responders and rescue team members during disaster response. To evaluate current research on resilience building interventions and their effectiveness in enhancing psychological resilience in these professionals. To highlight gaps in the existing literature and suggest directions for future research on supporting psychological resilience in first responders.

**Methods:**

A thorough literature search was conducted across several databases, including Web of Science, Scopus, Cochrane, PubMed, Medline, and Embase, focusing on articles published between 2019 and 2023. The search terms used were (emergency OR disaster) AND (psychological resilience) AND (rescue workers OR first responders OR firefighters OR ambulance personnel AND prehospital emergency medical services). Articles were selected based on inclusion and exclusion criteria, and only full-text articles published in English or Turkish were considered. Qualitative synthesis was used to analyze the data and draw insights.

**Results:**

The review reveals several critical factors affecting psychological resilience, including stress management techniques, emotional regulation strategies, social support systems, and training programs. It also identifies areas where existing research is lacking, particularly regarding specific interventions designed to bolster resilience in high-stress environments.

**Conclusions:**

Enhancing psychological resilience in first responders is crucial for both their individual well-being and the effectiveness of disaster response efforts. This review provides valuable insights into the factors that contribute to resilience and highlights the need for targeted interventions and support systems. By addressing these factors and promoting resilience, it is possible to improve both the performance of first responders and their capacity to cope with the demands of disaster situations.

**Keywords:**

Psychological resilience, first responders, rescue workers, mental health.

**Disclosure of Interest:**

None Declared

